# Simultaneous Remote Non-Invasive Blood Glucose and Lactate Measurements by Mid-Infrared Passive Spectroscopic Imaging

**DOI:** 10.3390/s25154537

**Published:** 2025-07-22

**Authors:** Ruka Kobashi, Daichi Anabuki, Hibiki Yano, Yuto Mukaihara, Akira Nishiyama, Kenji Wada, Akiko Nishimura, Ichiro Ishimaru

**Affiliations:** 1 Graduate School of Science for Creative Emergence, Kagawa University, 2217-20 Hayashi-cho, Takamatsu 761-0396, Kagawa, Japan; 2 Faculty of Medicine, Kagawa University, 1750-1 Miki-cho, Kita 761-0793, Kagawa, Japan; 3 Faculty of Engineering and Design, Kagawa University, 2217-20 Hayashi-cho, Takamatsu 761-0396, Kagawa, Japan

**Keywords:** non-invasive lactate measurement, non-invasive glucose measurement, simultaneous non-invasive measurements, mid-infrared passive spectroscopy

## Abstract

Mid-infrared passive spectroscopic imaging is a novel non-invasive and remote sensing method based on Planck’s law. It enables the acquisition of component-specific information from the human body by measuring naturally emitted thermal radiation in the mid-infrared region. Unlike active methods that require an external light source, our passive approach harnesses the body’s own emission, thereby enabling safe, long-term monitoring. In this study, we successfully demonstrated the simultaneous, non-invasive measurements of blood glucose and lactate levels of the human body using this method. The measurements, conducted over approximately 80 min, provided emittance data derived from mid-infrared passive spectroscopy that showed a temporal correlation with values obtained using conventional blood collection sensors. Furthermore, to evaluate localized metabolic changes, we performed k-means clustering analysis of the spectral data obtained from the upper arm. This enabled visualization of time-dependent lactate responses with spatial resolution. These results demonstrate the feasibility of multi-component monitoring without physical contact or biological sampling. The proposed technique holds promise for translation to medical diagnostics, continuous health monitoring, and sports medicine, in addition to facilitating the development of next-generation healthcare technologies.

## 1. Introduction

In recent years, there has been a growing interest in “biometric technology” for the quantitative evaluation of the health status of individuals to optimize exercise programs, rehabilitation, and even athletic performance [1]. In particular, quantitative evaluation of the metabolic response of the body to exercise is extremely important in sports science and the health management of older adults. For example, simultaneous monitoring of blood glucose and lactate provides important physiological indicators to comprehensively understand muscle energy metabolism, exercise intensity, fatigue level, and recovery state [2]. Blood glucose is used as an energy source to generate ATP mainly through aerobic metabolism at rest or during light exercise [3]. However, during high-intensity exercise or when oxygen supply is insufficient, the anaerobic glycolytic system is activated, and glucose is converted to pyruvate and then to lactate. This process results in a gradual decrease in the blood glucose level but an increase in the blood lactate level [4,5]. During the recovery period after exercise, lactate accumulated in the blood is resynthesized into glucose in the liver through the Cori cycle. This gradually restores the blood glucose level, and the blood lactate level begins to decrease [6,7]. Thus, blood glucose and lactate levels do not fluctuate unidirectionally. Instead, they have a dynamic metabolic relationship that reverses itself over time. Therefore, by observing both levels simultaneously and continuously, it is possible to obtain detailed overall information on metabolism that cannot be obtained with a single indicator. Furthermore, lactate is not necessarily produced as a metabolite of glucose only. For example, it is known that red blood cells and some anoxic metabolizing cells also produce lactate from other substrates such as fatty acids and amino acids [8,9,10]. In addition, a characteristic metabolism that converts glucose to lactate occurs in cancer cells even under aerobic conditions owing to the Warburg effect [11]. Thus, lactate is a metabolic intermediate that reflects a wide range of metabolic activities in the body, and measuring its level simultaneously with the blood glucose level provides a more comprehensive picture of metabolism. For example, early confirmation of an increase in the lactate level can be used to predict the point of transition to anaerobic metabolism and the onset of fatigue accumulation. Moreover, a rapid drop in the blood glucose level during exercise can signify hypoglycemia and energy depletion [12,13]. In addition, if lactate is not sufficiently metabolized and remains in the blood for a long period of time, then metabolic capacity may decrease, or recovery function may be impaired. These two indices can be applied, in combination, to evaluate the training and endurance of athletes or to assess the exercise tolerance of individuals suffering from a disease [14,15].

Currently, continuous glucose monitoring of these two indices requires capillary blood sampling or subcutaneous implanting of sensors for blood glucose and venous blood sampling for lactate, both of which are invasive and difficult to perform continuously [16,17]. Furthermore, there are limitations in capturing metabolic changes in real time during and immediately after exercise. In particular, evaluations in environments where exercise is performed continuously for long periods of time and medical settings require non-contact and immediate-response measurement methods [18]. To address these issues, this study examined the feasibility of non-invasive simultaneous measurements of blood glucose and lactate levels using mid-infrared passive spectroscopic imaging. This method can obtain information on each component by acquiring spectral characteristics from radiated light in the mid-infrared region emitted from the human body. We have already demonstrated that the proposed method can remotely measure the blood glucose level, which represents the concentration of glucose in the human body [19]. It has also been shown that the emission integral effect enables the measurement of glucose at depths of 1–4 mm in the human body [20]. Furthermore, lactate also has a specific emission peak in the mid-infrared region; it is possible to simultaneously observe the variations of both components spatially and temporally in the same field of view. Thus, this study aimed to establish a new non-invasive monitoring technique to visualize the entire metabolic networks of blood glucose and lactate in real time, demonstrating its high potential for translation to the fields of exercise physiology, medicine, and rehabilitation.

## 2. Materials and Methods

### 2.1. Principle of Apparatus

Active spectroscopic methods such as Fourier-transform infrared spectroscopy (FTIR), which is widely used in mid-infrared spectroscopy, require an infrared light source such as a kanthal light source to irradiate the measurement target, and the molecular vibration excited by the light is measured. The spectral characteristics are obtained as an absorption spectrum in which the energy corresponding to the eigenfrequency of the molecule is absorbed [21]. In contrast, our proposed mid-infrared passive spectroscopic imaging obtains spectral characteristics by measuring the spectral radiance of infrared radiated light emitted by the measurement target. Radiated light is emitted with an intensity that depends on the temperature based on Planck’s law, and spectral characteristics can be obtained at wavelengths corresponding to the eigenfrequencies of the molecules. Therefore, light absorption is observed in active spectroscopy, and molecular vibration can be observed as a radiation phenomenon in passive spectroscopy at the same wavelength.

### 2.2. Internal Configuration of Optics

**Figure 1:**
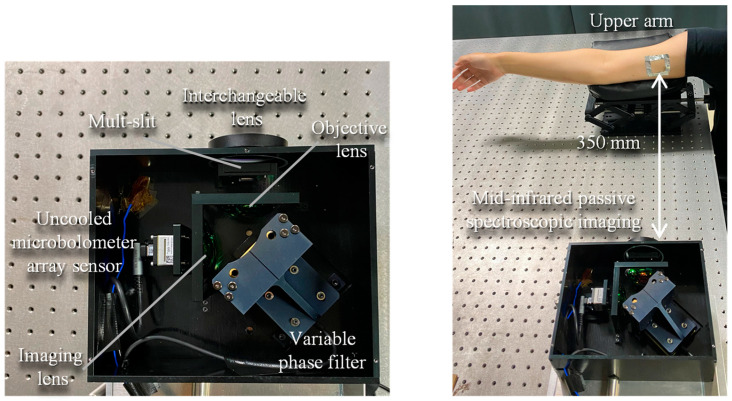
Internal configuration of the mid-infrared passive spectroscopic imager and experimental optics for upper arm measurements using the proposed method.

Figure 1 shows the experimental optical system used to measure the upper arm by mid-infrared passive spectroscopic imaging. The system consists of the following components in an infinite conjugate ratio design: an objective lens (germanium, 50 mm lens diameter, 25 mm focal length), an imaging lens (germanium, 50 mm lens diameter, 25 mm focal length), a variable phase filter, and an uncooled microbolometer array sensor (array size: 320 × 256 pixels, pixel pitch: 12 µm, sensitivity range: 7.5 to 13.5 µm, Boson 320; FLIR Systems, Wilsonville, OR, USA) [22,23]. The variable phase filter, consisting of a fixed mirror and a movable mirror, is an associate common-path phase-shift interferometer, which makes the optical system highly robust to mechanical vibration. Furthermore, a multi-slit is placed on the conjugate plane of the detector to improve the clarity of interference fringes and prevent bright spots from canceling out [24]. This enables stable spectroscopic measurements even of weak infrared radiated light emitted from the measurement target at room temperature.

### 2.3. Measurement of Absorbance and Emittance of Lactic Acid

In this experiment, the upper arm of one adult male participant (over 18 years old) was measured at a distance of 350 mm from the device, as shown in Figure 1. The absorbance was obtained by measuring a solution of lactic acid (2000 mg/dL) by FTIR, and the emittance was obtained by measuring the participant’s upper arm by mid-infrared passive spectroscopic imaging.

### 2.4. Blood Lactate Measurement

Using the proposed method, we measured the upper arm at intervals of 10 min from 10 min (start of measurement) to 60 min (end of measurement). The period of 0 min was used as the background. In parallel with each measurement, a blood-collecting lactate sensor (Lactate Pro 2 LT-1730, Arkray, Kyoto, Japan) was used to measure the lactate level. The proposed method measured the right upper arm, and the blood-collecting lactate sensor measured the index finger of the right hand. After 10 min of measurement, the subject exercised with dumbbells to increase the lactate level of the right upper arm, the measurement site [25,26]. Furthermore, to prevent misalignment of the measurement site over time, the upper arm was marked with low-emissivity aluminum tape. This allowed us to compare the time trends of the emittance and lactate values. In addition, to minimize the effect of perspiration on the change in emittance, the measurement sites were wiped with alcohol swabs before each measurement.

### 2.5. Measurement of Blood Lactate and Blood Glucose Levels

In the measurements of blood lactate and blood glucose levels, the measurement time was extended to 80 min. In addition to the proposed method, a blood lactate sensor and a blood glucose sensor (GLUCOCARD PlusCare, Arkray, Kyoto, Japan) were used to measure lactate and glucose levels, respectively. In the proposed method, the right upper arm was used for lactate measurement and the right wrist for blood glucose measurement. The two blood-collecting sensors measured the index finger of the right hand. Immediately after the start of measurement, the participant exercised with dumbbells for 10 min to increase the lactate level in the right upper arm. After 40 min of measurement, the participant consumed a drink containing sugar to induce an increase in the blood glucose level. In addition, aluminum tape was applied to the right upper arm and right wrist as a position marker to prevent misalignment of the measurement site over time. In this experiment, lactate and blood glucose were measured alternately. However, the time between each measurement was very short, approximately 10 s. Metabolic components such as glucose and lactate usually undergo concentration changes on a time scale of several minutes to several tens of minutes. Therefore, it can be concluded that the time difference of 10 s has practically no effect on the evaluation of metabolic kinetics [27,28]. For this reason, the method used in this study can be regarded as a simultaneous measurement.

### 2.6. Visualization of Time Response of Lactic Acid by k-Means Method

The time response of lactate was visualized by analyzing the lactate emittance data in the human body obtained by the proposed method using k-means clustering, an unsupervised learning method. The k-means method is an algorithm that divides data into a predefined number of clusters and classifies data points within each cluster so that they are closest to the center, and it is effective for extracting regions showing similar time-series changes [29]. In this analysis, the number of clusters was set to 3 on the assumption that the time response of lactate could be classified into three types. Regions with high interference clarity were selected for the analysis. The data used for the analysis consisted of emittance values at the peak wavelength of lactate (8.9 µm) acquired at intervals of 10 min from 10 min to 60 min, for a total of six time points. Therefore, the data consisted of six-dimensional data with the emittance change on the time axis at each pixel as the feature value, and this temporal transition was used as the main axis of clustering. To eliminate absolute differences between different pixels and to focus on relative temporal changes only, normalization was applied to all emittance data.

## 3. Results

### 3.1. Non-Invasive Lactate Measurement of the Upper Arm

**Figure 2:**
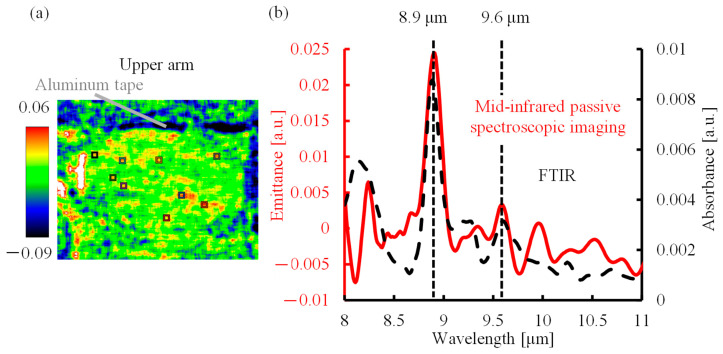
Mid-infrared passive spectroscopic imaging of the upper arm. (**a**) Emittance map of the upper arm at 8.9 µm. (**b**) Absorption spectrum of lactic acid solution and emission spectrum of the upper arm.

The absorption spectrum of the lactic acid solution measured by FTIR (Figure 2b, black dotted line) confirmed that the characteristic absorption peaks of lactic acid were located at the wavelengths of 8.9 and 9.6 µm. Therefore, an emittance map was created to show the emittance of lactic acid at the peak wavelength of 8.9 µm, as shown in Figure 2a. The emittance was calculated for the average spectrum of nine areas (9 × 9 pixels, squares in the emittance map) using the emission spectrum of a blackbody, and the result is the solid red line in Figure 2b. The merged area was set to 9 × 9 pixels, as required by the structure of the multi-slit. A comparison of the emission spectrum with the absorption spectrum revealed that the emission peaks at the wavelengths of 8.9 and 9.6 µm coincided with the absorption peaks of lactic acid (black dotted line). This shows that the proposed method can be used for non-invasive measurements of lactic acid in the human body.

### 3.2. Lactic Acid in the Human Body over Time

**Figure 3:**
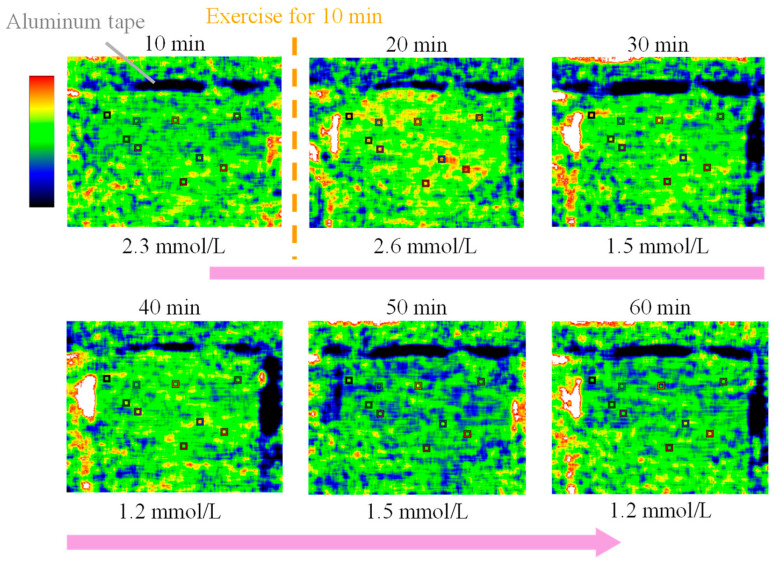
Emittance map at the peak wavelength of lactate (8.9 µm) measured every 10 min from 10 to 60 min showing the time variation of the blood lactate level.

Figure 3 shows the emittance map at the wavelength of 8.9 µm. Areas of high emissivity are shown in yellow or red, and areas of low emissivity are shown in blue. The values in the upper part of the image show the elapsed time, and the values in the lower part of the image show the lactate level measured in parallel using a blood-collecting lactate sensor. The lactate level at the beginning of the measurement (10 min) was 2.3 mmol/L, and it increased to 2.6 mmol/L after exercise (20 min). Furthermore, the lactate level decreased to 1.5 mmol/L and 1.2 mmol/L at 30 and 40 min (after exercise), respectively. This change was also observed in the emittance map, which showed the appearance of high-emissivity yellow and red immediately after exercise, followed by a shift to low-emissivity blue. Therefore, mid-infrared passive spectroscopic imaging has the potential to remotely monitor temporal changes in the lactate level. In addition, the emittance map of the upper arm, with many muscles, was particularly bright after exercise, indicating the possibility of visualizing lactate accumulation in the human body using the proposed method.

**Figure 4:**
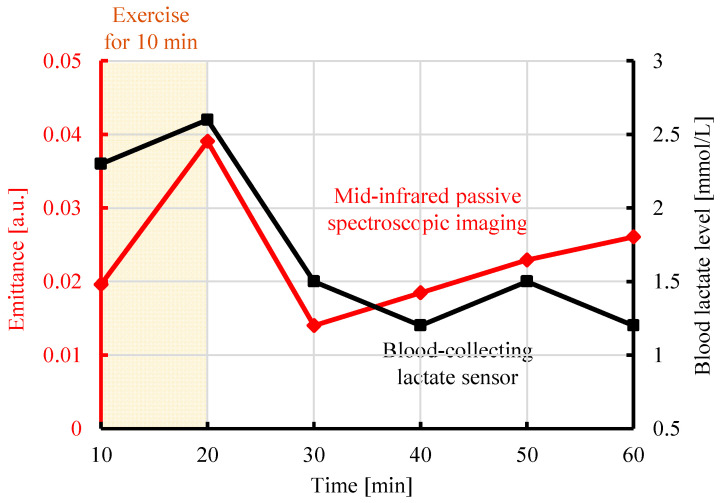
Comparison of the emittance at 8.9 µm obtained from mid-infrared passive spectroscopic imaging of the upper arm and the blood lactate level measured by a blood-collecting lactate sensor.

The time variations of the lactate level and emittance (8.9 µm) measured by the blood-collecting lactate sensor and proposed method, respectively, are shown in Figure 4. The black line shows the blood lactate level [mmol/L] measured by the blood-collecting lactate sensor as a function of time. The lactate level increased after 10 min of exercise, then decreased, and returned to the baseline. The red line shows the emittance [a.u.] at 8.9 µm, the peak wavelength of lactate as a function of time. The analysis points were selected by referring to the emittance map, shown in Figure 3, and identifying regions in which lactate exhibited a high time response, changing to yellow or red after exercise. Similarly, the emittance of lactate increased after exercise, then decreased, and returned to the baseline. This correspondence can be explained on the basis of molecular-level mechanisms. Radiated light is generated by molecular vibrations corresponding to the natural frequencies of molecules. As the concentration of lactate increases in a tissue, the number of lactate molecules increases and the intensity of radiated light at the corresponding wavelength also increases. Therefore, the emittance increases as the lactate concentration increases [21]. Therefore, the emittance from mid-infrared passive spectroscopic imaging correlated with the lactate level from the blood-collecting lactate sensor, and the transition of the emittance due to exercise was quantitatively confirmed.

### 3.3. Simultaneous Non-Invasive Detection of Blood Glucose and Lactate in the Human Body

**Figure 5:**
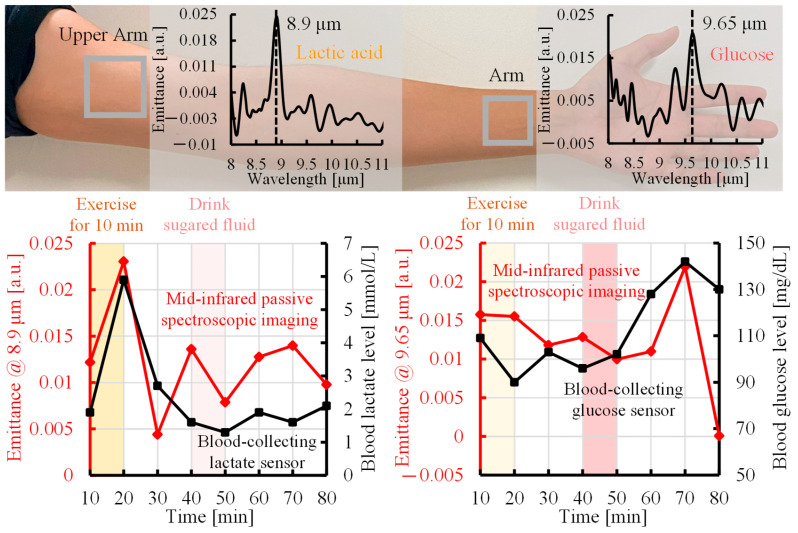
Comparison of time trends of the emittance measured by mid-infrared passive spectroscopic imaging with lactate and blood glucose levels from blood-collecting sensors.

As shown in the upper panel of Figure 5, the upper arm emitted light with a peak wavelength of 8.9 µm, which corresponded to lactate because of the large distribution of muscles. In contrast, the wrist emitted light with a peak wavelength of 9.65 µm, which was attributed to glucose because of the large blood vessels located close to the body surface. Consequently, we analyzed blood lactate and glucose separately at the upper arm and wrist, respectively. Figure 5 shows the experimental results measured by the proposed method, a blood-collecting lactate sensor, and a blood-collecting blood glucose sensor. The lower left panel of Figure 5 shows the lactate emittance measured by the proposed method (red line) and the blood lactate level [mmol/L] measured by the blood-collecting lactate sensor (black line). As shown in Figure 4, regions in which lactate exhibited a high time response were selected for analysis. The lactate level increased after 10 min of exercise, then decreased, and returned to the baseline. The emittance at 8.9 µm, the peak wavelength of lactate, increased after exercise, then decreased, and returned to the baseline, consistent with the lactate level. In addition, the lower right-hand panel of Figure 5 shows the time variation of the glucose emittance measured by the proposed method (red line) and time variation of the blood glucose level [mg/dL] measured by the blood glucose sensor (black line). The blood glucose level did not change after exercise, although it increased after sugar intake and then decreased. The red line shows the change in emittance at 9.65 µm, the peak wavelength of glucose. Similarly, the emittance did not change after exercise, and it increased after the ingestion of glucose and then decreased. These results confirmed the feasibility of simultaneously detecting blood glucose and lactate levels non-invasively by mid-infrared passive spectroscopic imaging.

## 4. Discussion

### 4.1. Discrepancy Between Mid-Infrared Passive Spectroscopic Imaging and Blood Collection-Type Sensors over Time

Figure 5 shows the existence of a correlation between the changes in emittance using the proposed method and the changes in blood concentration using the conventional method. However, a discrepancy was observed in the lactate data after 30 min from the start of the measurement and in the glucose data at 80 min from the start of the measurement. One of the reasons for this discrepancy is the difference in the measurement site. The proposed method acquires spectral characteristics of components in subcutaneous tissue through the surface of body parts, such as the upper arm and wrist, and reflects the state of the component in the local tissue as a whole. In contrast, the blood-collecting sensor measures only changes in the concentration of the component in peripheral blood collected from the fingertip. Therefore, there are differences in the distribution of tissues and measured components between the proposed method and the blood-collecting sensor, and the differences in local metabolism and the characteristics reflected in blood may result in discrepancies in the data, especially in the lactate level after 30 min and in the glucose level at 80 min.

### 4.2. Verification of Time Response of Lactate in Human Body

**Figure 6:**
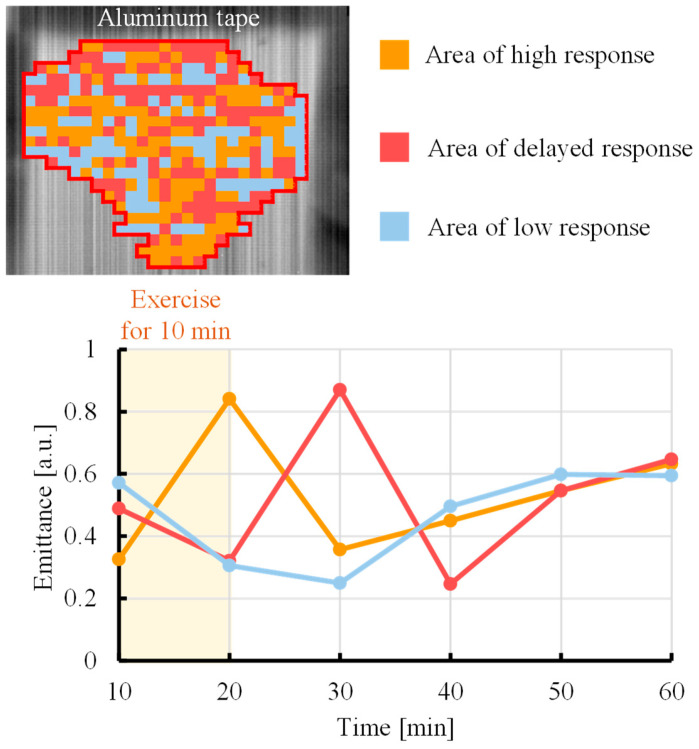
Response of lactate to exercise over time was visualized in the upper arm by cluster analysis using the k-means method.

During exercise, lactate is produced in skeletal muscles and then released into blood vessels via monocarboxylate transporters in cell membranes [30]. As a result, the blood lactate concentration increases, and lactate circulates throughout the body. However, there is a time lag between the production of lactate in muscles and fluctuations in the blood lactate level. Specifically, lactate is rapidly produced in skeletal muscles immediately after exercise but requires a certain amount of time to be transferred to blood vessels. Figure 6 shows the results of k-means spatial classification of the lactate emittance data obtained by the proposed method, allowing visualization of the response of lactate to exercise over time. The lower graph shows the average emittance of all pixels in each cluster over time. The orange region is the area where the emittance increases immediately after exercise, indicating the rapid production and accumulation of lactate in response to exercise. In the red region, the emittance increases sometime after exercise, indicating a delayed accumulation of lactate owing to diffusion and migration. In addition, light blue marks a region of low responsiveness to exercise. These results indicate that k-means cluster analysis can be used to visualize lactate generation and diffusion from temporal changes in the emissivity. The emittance maps (Figure 3) can visualize quantitative information, the spatial accumulation of lactate, which is the concentration of lactate at a specific point in time, based on the intensity distribution of emittance. In contrast, k-means clustering (Figure 6) can visualize the dynamic information of the generation and diffusion process of lactate along the temporal phase information. This study is the first to attempt an analysis of lactate dynamics using image data with temporal resolution. Regions in which lactate exhibited a high time response (Figure 4 and Figure 5) were selected for analysis. However, if other regions are selected, as shown in Figure 6, then changes in the lactate level may exhibit a time delay compared with the trend observed using a blood-collecting sensor.

## 5. Conclusions

In this study, we demonstrated that the levels of blood lactate and glucose in the human body can be measured non-invasively and simultaneously using mid-infrared passive spectroscopic imaging. Using the proposed method, we detected lactate-induced light emission at 8.9 µm from the upper arm, the measurement target, and continuously measured the emittance at 8.9 µm to visualize the lactate level over time. In addition, a correlation was found to exist between changes in the emittance and fluctuations in the lactate level, which was determined with a conventional blood-collecting lactate sensor, confirming the possibility of non-contact, non-invasive remote monitoring of the lactate level using our method. Furthermore, lactate and glucose (9.65 µm) emission peaks were extracted from experimental data of the upper arm and wrist, respectively, demonstrating that both components can be measured simultaneously and non-invasively. The response of lactate in the upper arm to exercise over time was spatially classified and visualized by k-means cluster analysis. Therefore, the proposed method has the potential to analyze and evaluate the effect of exercise on athletes by monitoring muscle metabolism during exercise loading and recovery. Future research should compare the above results with those obtained after analysis using other clustering methods. The validity of the classification results obtained in Figure 6 and the reliability of the temporal features will be further verified through animal experiments. Furthermore, while the intensities of lactate and glucose emissions in Figure 5 appear similar, the actual blood concentrations of lactate and glucose are different [31,32]. Previous studies have shown that the emittance depends on the concentration and absorption coefficient as well as the depth at which the component exists [20]. The similar intensities of lactate and glucose emissions in this study are considered to arise from the combined effects of these multiple factors. However, because the absorption coefficient of lactic acid has not been elucidated at this point, detailed verification of the optical properties of lactic acid and calibration through animal experiments are necessary to accurately evaluate and clarify the quantitative relationship between the emittance and concentration of the components. Mid-infrared passive spectroscopic imaging has the potential to simultaneously and non-invasively acquire information on multiple components in the human body. In the future, this device is expected to be installed in homes and sports facilities to support personalized training and safe exercise guidance for older adults through real-time evaluation of an individual’s exercise response and fatigue recovery.

## Data Availability

The data presented in this study are available upon request from the corresponding author.
